# La hernie diaphragmatique congénitale: une pathologie pas toujours facile à diagnostiquer

**DOI:** 10.11604/pamj.2020.36.353.13525

**Published:** 2020-08-27

**Authors:** Soukaina Ait Hmadouch, Amina Barkat

**Affiliations:** 1 Service de Néonatologie P5, Hôpital d’Enfant, CHU Ibn Sina, Rabat, Maroc,; 2 Equipe de Recherche en Santé et en Nutrition du Couple Mère-enfant, Faculté de Médecine et de Pharmacie de Rabat, Université Mohamed V, Rabat, Maroc

**Keywords:** Hernie, diaphragme, diagnostic anténatal, Hernia, diaphragm, antenatal diagnosis

## Abstract

La hernie congénitale de la coupole diaphragmatique (HCCD) est une embryopathie congénitale qui se définit par l’absence de développement de tout ou d’une partie d’une coupole diaphragmatique. La fréquence de cette pathologie est de l’ordre de 1/3500 naissances vivantes avec une prédominance masculine. Ce travail porte sur l’étude d’un cas de hernie de Bochdalek, ainsi que les données de littérature et consiste à décrire les difficultés diagnostiques et thérapeutiques rencontrées devant cette pathologie. Le diagnostic et la prise en charge anténatale sont des éléments importants pour réduire la mortalité et la morbidité des patients. Le pronostic demeure encore sévère et reste tributaire de l’existence et du degré de l’hypoplasie pulmonaire et de l’association à des malformations congénitales.

## Abstract (en)

Congenital hernia of the diaphragmatic dome (CDC) is a congenital embryopathy where all or part of the diaphragmatic dome fails to form. Its rate is 1/3500 live births with a male predominance. This study reports a case of Bochdalek hernia as well as data in the literature and highlights the diagnostic and therapeutic challenges in this disease. Diagnosis and antenatal management are essential to reduce patient’s mortality and morbidity. Prognosis is poor and depends on the presence and degree of pulmonary hypoplasia and on the association with congenital malformations.

## Introduction

La hernie diaphragmatique congénitale (HDC) se définit par l’absence de développement de tout ou d’une partie d’une coupole diaphragmatique, cette anomalie entraîne la présence dans le thorax de certains viscères abdominaux aux moments cruciaux du développement pulmonaire fœtale [1]. Dans 80% des cas il s’agit de la partie postéro-latérale de la coupole gauche appelée hernie de Bochdalek [1,2]. Le diagnostic de la hernie diaphragmatique repose essentiellement sur la radiographie pulmonaire standard mais dans certain cas il faut avoir recours à d’autres examens pour confirmer le diagnostic. Les HDC sont soit isolées, soit associées à d’autres malformations (hypoplasie pulmonaire, HTAP, PCA, FO, malrotation intestinale, diverticule de Meckel et/ou des anomalies chromosomiques (trisomie 13,18) [2,3]. À la lumière d’une observation d’un cas et de la littérature, l’objectif de ce travail est de marquer le point sur les difficultés diagnostiques et la prise en charge de cette pathologie.

## Patient et observation

**Figure 1:**
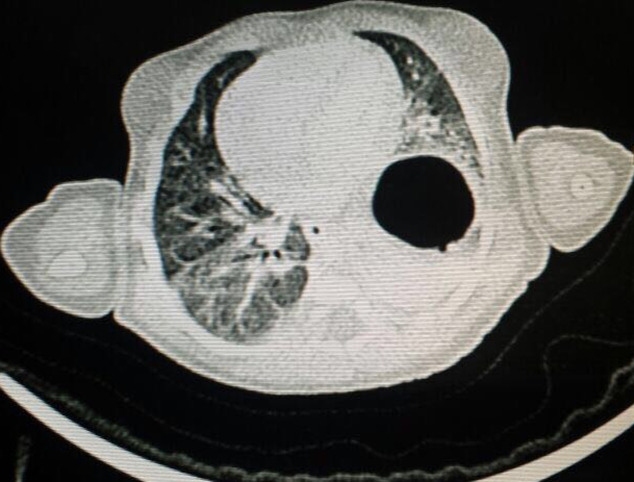
coupe transversale montrant une hernie diaphragmatique de Bochdalek

Le patient étudié était un nouveau-né de sexe féminin, admis à J4 de vie. La mère était âgée de 26 ans, G2P2, sans antécédents notables avec absence de consanguinité. La grossesse a été mal suivie et l’accouchement s’était déroulé par voie basse au Centre Hospitalier Provincial (CHP) de Tanger avec bonne adaptation à la vie extra-utérine. Le nouveau-né avait été remis à sa mère avec apparition d’une DR 2/10 à J2 de vie, d’où son transfert. À l’admission, le nouveau-né était polypnéique et à l’examen on avait noté une détresse respiratoire 1-2/10 avec abolition des murmures vésiculaires à gauche avec plusieurs tubercules prétragiens du côté droit et une masse mobile indolore d’aspect mamelonnée au niveau du crâne. La radiographie du thorax montrait un niveau hydro-aérique en intra thoracique. On avait hospitalisé le malade sous lunette d’oxygène avec traitement anti-reflux. Un bilan biologique a été réalisé qui est revenue sans particularité et l’évolution a été marquée par la disparition de la détresse respiratoire après 24h d’hospitalisation, ce qui avait posé un problème diagnostique entre hernie diaphragmatique et hernie hiatale congénitale. Un transit œso-gastro-duodénal (TOGD) avait été réalisé qui montrait l’estomac en position intra thoracique. La tomodensitométrie (TDM) thoracique avait objectivé une hernie diaphragmatique de Bochdalek (Figure 1). Dans le cadre du bilan malformatif, l’échographie abdomino-rénale était normale, alors que l’échographie transfontanellaire (ETF) montrait un kyste épidermoïde au niveau du 4^e^ ventricule. Le malade a été présenté aux chirurgiens avec rendez-vous pour intervention chirurgicale dans les semaines à suivre.

## Discussion

Dans la littérature l’incidence rapportée de la hernie diaphragmatique congénitale varie entre 1/3000 à 1/5000 naissances vivantes [1,4,5]. La plupart des études rapportent une prédominance masculine nette dans la hernie diaphragmatique congénitale (HDC): elle survient de 30 à 50% plus chez les garçons que chez les filles [4,6,7]. Dans notre cas, le patient est de sexe féminin. L’âge des patients varie entre 3 mois et 11 mois [4]. L’atteinte diaphragmatique est habituellement unilatérale, avec une prédilection pour le côté gauche dans près de 80 à 90% des cas et dans 10 à 15% des cas à droite, elle est exceptionnellement bilatérale moins de 1% [1,2,4,6]. Ceci pourrait s’expliquer par une fermeture plus précoce du canal pleuro-péritonéal droit ou par la présence hépatique [8]. La HDC associe souvent: une hypoplasie pulmonaire, des anomalies du lit vasculaire pulmonaire anatomiques (réduction du nombre de vaisseaux et hypermuscularisation des artérioles) et fonctionnelles (déséquilibre entre médiateurs vasodilatateur et vasoconstricteur) et un hypodéveloppement du cœur gauche. La présence, ainsi que l’importance de ces malformations augmentent la gravité de la pathologie et justifie une prise en charge planifiée dans un centre néonatal [2,9]. La hernie de Bochdalek à révélation néonatale est mal tolérée du fait de l’hypoplasie pulmonaire associée (due à la pression prolongée in utero des viscères abdominaux herniés dans le thorax) à l’origine d’une hypoxémie et d’une acidose. L’hypertension artérielle pulmonaire (HTAP) qui en résulte crée un shunt gauche-droit qui majore et entretient le processus. Le tableau est en général bruyant avec une DR aigüe qui nécessite une intervention chirurgicale en urgence [10], alors que notre malade avait bien supporté cette malformation ce qui a posé un problème de diagnostic, d’où le recours à la TDM thoracique. Les HDC de révélation tardive représentent 5 à 30% des cas [11]. Ce retard diagnostique est dû à la rareté de cette pathologie, ainsi qu’à une sémiologie trompeuse et variée, associant ou non des signes respiratoires, digestifs et parfois infectieux [12]. Elle pose un problème de diagnostic différentiel avec de nombreuses pathologies: maladie adénomatoïde du poumon, séquestre pulmonaire, tératome kystique, tumeur neurologique, tumeur para-œsophagienne agénésie pulmonaire... Dans une étude antérieure, jusqu’à un tiers des cas d’HDC chez des nouveau-nés ont d’abord été mal diagnostiqués et seulement 29% des bébés ont été diagnostiqués dans les 24heures suivant la naissance [13,14]. La physiopathologie du retard d’expression de la HDC est mal connue. L’obstruction de l’orifice herniaire diaphragmatique par certains organes abdominaux tels que le foie ou la rate pourrait expliquer le délai d’apparition des signes [11]. La pathologie peut se révéler à l’occasion d’une élévation brusque de la pression abdominale (toux, effort, vomissement, traumatisme) [11]. Dans ces cas de HDC de révélation tardive, la localisation postérolatérale gauche reste la plus fréquente (hernie de Bochdalek) [15].

Notre malade avait une association entre hernie diaphragmatique et kyste épidermoïde cérébrale. Les kystes épidermoïdes sont des formations tumorales bénignes, de croissance lente, presque toujours d’origine congénitale, résultant de l’inclusion aberrante d’éléments ectodermiques, lors de la fermeture du tube neural, entre la 3^e^ et la 5^e^ semaine du développement embryonnaire. La formation du diaphragme se déroule en 2 étapes: le diaphragme primitif doit son existence au développement des sacs cœlomiques de l’embryon. Cette étape se déroule de la fin de la 3^e^ jusqu’au début de la 4^e^ semaine de gestation. À la fin de cette phase, le diaphragme primitif est constitué ventralement du septum transverse et dorsalement des canaux pleuro péritonéaux ainsi que leurs structures environnantes (membrane pleuro péritonéale et médiastin) [16,17]. La seconde étape se déroule de la 4^e^ à la 8^e^ semaine de gestation dont, cependant, les processus ne sont pas très bien connus. L’association entre ces 2 malformations reste non connue mais ils résultent tous les deux d’une anomalie de formation lors des premières semaines de gestation. Le traitement radical est chirurgical, soit par voie transversale sus ombilicale au niveau de la pointe des 10^e^ côtés, soit par voie médiane sus ombilicale. La hernie diaphragmatique congénitale est une pathologie très grave, elle présente une mortalité assez importante. La mortalité est de 48% dans les HDC isolées; l’association à une malformation ou la prématurité aggrave le pronostic avec une mortalité de 80% et 83% respectivement [10]. Grâce aux progrès du diagnostic prénatal et de prise en charge pluridisciplinaire précoce, la survie est de 80% [18,19].

## Conclusion

La hernie diaphragmatique congénitale est une pathologie rare, mais grave avec une mortalité très élevée. Le diagnostic, vu le polymorphisme sémiologique de cette maladie et ses différents degrés de gravité, reste un gros problème et peut impacter le pronostic et la prise en charge ultérieure. De ce fait, il faut toujours suspecter la HDC devant une détresse respiratoire et une imagerie évocatrice. Plusieurs associations avec d’autres malformations ont été décrites et leur découverte peut guider le diagnostic.
